# Laser-accelerated particle beams for stress testing of materials

**DOI:** 10.1038/s41467-017-02675-x

**Published:** 2018-01-25

**Authors:** M. Barberio, M. Scisciò, S. Vallières, F. Cardelli, S. N. Chen, G. Famulari, T. Gangolf, G. Revet, A. Schiavi, M. Senzacqua, P. Antici

**Affiliations:** 1 INRS-EMT, 1650 Boul. Lionel Boulet, Varennes, QC Canada; 2 University of Rome “La Sapienza”, Dip. SBAI and INFN, Via A. Scarpa 16, 00161 Roma, Italy; 3 LULI, Ecole Polytechnique, Route de Saclay, 91128 Palaiseau, France; 4 Institute of Applied Physics, 46 Ulyanov Street, Nizhny Novgorod, Russia 603950; 5 Medical Physics Unit, McGill University, Montreal, QC Canada

## Abstract

Laser-driven particle acceleration, obtained by irradiation of a solid target using an ultra-intense (*I* > 10^18^ W/cm^2^) short-pulse (duration <1 ps) laser, is a growing field of interest, in particular for its manifold potential applications in different domains. Here, we provide experimental evidence that laser-generated particles, in particular protons, can be used for stress testing materials and are particularly suited for identifying materials to be used in harsh conditions. We show that these laser-generated protons can produce, in a very short time scale, a strong mechanical and thermal damage, that, given the short irradiation time, does not allow for recovery of the material. We confirm this by analyzing changes in the mechanical, optical, electrical, and morphological properties of five materials of interest to be used in harsh conditions.

## Abstract (web summary)

Recently, there has been significant progress on the application of laser-generated proton beams in material science. Here the authors demonstrate the benefit of employing such beams in stress testing different materials by examining their mechanical, optical, electrical, and morphological properties.

## Introduction

In the past decade, intense research has been conducted on the topic of laser-accelerated particle beams, produced during the interaction of a solid target with a high intensity (*I* > 1 × 10^18^ W/cm^2^), short-pulse (<1 ps) laser. Today, routinely obtained laser-generated particles, in particular protons, exhibit about 10^13^ particles per shot, have a ps long duration at the source, have an energy in the tens of MeV range^1^ and very good laminarity^2^. While strong effort is put to materialize different applications, such as in astrophysics^3,4^, bright ultra-short neutron sources^5,6^, medicine^7^, or injectors for large-scale accelerators^8,9^, material science applications are still in a very embryonic state with some interesting pioneering works presented recently^10–12^. Conversely, laser-driven protons can offer many opportunities in this field^13^, in particular when benefitting from their high particle flux that provides ideal conditions for performing and analyzing stress tests on different materials that are exposed to high-energy fluence, i.e., harsh conditions. Examples of these conditions can be found in high-energy density physics, astrophysics, aero spatial applications, or energy production^14^ (nuclear plants, but also upcoming facilities for inertial or magnetic confinement fusion (ICF-MCF)^15^ in particular for their plasma-facing materials (PFM)^16–18^). Currently, three stress test methods are the most commonly employed. These include electron beam simulation of disruption heat flux, He or Gamma-ray beam irradiation, and exposure to a laboratory He plasma. All these methods give information on the changes of the material properties, but only the combination of all methods can  provide a complete analysis of the material response to stress. Moreover, these tests require long exposure times, are extremely complex to model computationally, and are unable to reproduce the real operational environment. Therefore, these tests suffer of many challenges that still need to be solved^19,20^.

In this paper, we provide experimental evidence that laser-generated protons can be used to perform and analyze stress tests on different materials. Compared to the existing methods, this laser-driven analysis has the advantage of being much faster, since it can be performed with a few single laser shots, and of being more compact, since it can be performed using a table-top high-power laser. We confirm this by testing the morphological, mechanical, electrical, and optical response of five materials. Stress testing occurs in several domains and industrial applications. In the present manuscript, we concentrate more on high-melting point materials typically employed in ICF-MCF facilities (and in particular as PFM), since there is a strong demand to improve materials on these facilities and hence there have been extensive studies related to them. As such, we focus in this study particularly on tungsten, a material currently used for typical ICF facilities or reactors, on carbon (graphite), the material currently used for divertors, secondary walls and junctions, and on three materials (titanium, tantalum, and molybdenum) suggested by literature as good candidates for realizing nano- or W-based composite structures, since having a melting point higher than the maximum working temperature required by PFM safety regulations^21^. We demonstrate that our laser-generated proton beam allows reproducing an equivalent damage to the material, as obtained normally only after several months of full operation of facilities producing a harsh environment for materials (e.g., ICF facilities or nuclear reactors). This equivalent damage to the material is produced by the fact that   the short and intense proton irradiation does not allow for recovery of the material.

## Results

### Experimental setup

**Fig. 1:**
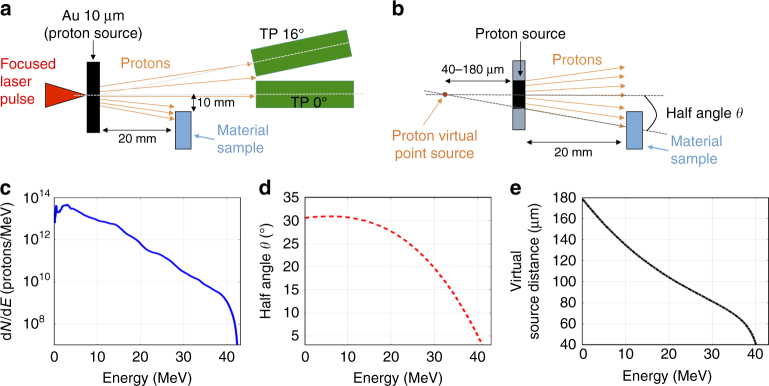
Experimental setup and proton source characteristics. **a** Experimental setup in optimal irradiation conditions; **b** Sketch of the source characterization with description of the virtual point source and the cone half angle *θ*. These parameters have been used in the Monte Carlo simulations; The virtual point source is not a physical source point, but its distance is calculated by the projection of the proton trajectories stemming out from the back surface of the target; **c** Example of a typical experimental proton spectrum obtained on the TITAN facility with the TP0°; **d** Half angle divergence (*θ*) vs. proton beam energy; **e** Virtual source point distance against proton beam energy

The experiments were performed on the TITAN laser facility located at the Lawrence Livermore National Laboratory (LLNL, USA)^22^. The experimental setup is shown in Fig. 1a. A laser with energy 180 J (<10% shot-to-shot energy fluctuations), pulse duration of *τ* = 700 fs, wavelength  *λ* = 1.053 µm and beam diameter 25 cm was focused down by an f/3 parabola (focal distance about 75 cm) under high vacuum conditions to a 9 µm focal spot diameter (full-width-half-maximum, FWHM), generating an intensity of *I* ~ 4 × 10^19^ W/cm^2^. The laser was hitting with normal incidence onto a 10 µm-thick gold foil (gold purity 99.9%, commercially available from the supplier Goodfellow) in order to accelerate protons in the laser-forward direction using the target-normal-sheath-acceleration (TNSA)^23^ mechanism. In this acceleration process, the focused laser pulse generates at the front surface, resulting from the ponderomotive force, energetic (hot) electrons with a mean energy of a few MeVs that travel through the target. While some electrons escape the target at its rear surface, most electrons are retained by the negatively charged bulk of the target and form at the rear target surface a dense electron sheath over a distance comparable to the Debye length (in this case about 1 µm). This creates at the back surface a charge separation electric field on the order of TV/m that accelerates residual water contaminants (mainly hydrogen) located on the initially unperturbed rear side of the target (the acceleration occurs in a timeframe shorter than the typical relaxation time of the bulk of the target). The ion beam is therefore accelerated normally from the rear surface of the laser-irradiated target.

The laser pulse was linearly S-polarized and the prepulse-to-main pulse contrast ratio was about 10^−6^, as it is typical for this class of laser systems. The repetition rate of the laser system is about one shot every 30 min, which corresponds to the waiting time needed to cool down the optical amplifiers.

We performed several shots varying the distance between the proton source and the material samples to be stress tested (distance ranging from 5 mm to 4 cm) in order to find the optimal distance. To allow for a measurement of the protons spectrum during the shot, the samples were placed 1 cm transversally above the beam center. A distance of 2 cm between the source and this transversally positioned material sample  (see Fig. 1a, b) was found to be the best compromise for avoiding a temperature within the sample above the melting point (obtained for a too short distance), yet irradiating the sample with a sufficiently high proton density (increasing the distance leads to a lower proton flux on the sample). Temperature maps of the samples, computed using a Monte Carlo code into which we inserted the proton source parameters, can be found in the Supplementary Fig. 3 and are discussed later. The temperature produced by the impinging protons was also monitored by placing materials with a known melting point (e.g., gold, melting around 1065 °C) inside the proton beam, and verifying that a melting process was taking place at the distance for which the code was predicting these temperatures. By fixing the distance at 2 cm, we ensured that there was no interaction between the sample and the secondary electrons emitted by the laser-plasma source (the threshold distance for avoiding this effect is typically in the range of a few hundreds of µm^24^).

For the material targets to be irradiated by the laser-generated protons, we used a series of commercially available solid targets with dimensions of about 2 × 20 mm and thickness of 500 µm. As proton beam diagnostics, we used two calibrated Thomson-Parabolas located at 0° (TP0°) and 16° (TP16°) with respect to the main laser pulse axis (see details in the Supplementary Note 2). The material–sample targets were placed occupying only half of the proton beam, so that the TP could read out the spectrum during each shot using the other half of the proton beam. The TPs also allowed measuring other ions that stem out of the rear target surface during the acceleration process (for a detailed spectrum of all emitted ions, see Supplementary Fig. 2). The maximum proton energy that was detected, exceeding 40 MeV, is in agreement with the maximum proton energy found on similar laser facilities^25,27^ or predicted by scaling law studies^28,29^. The stability of the laser system allowed to achieve a good repeatability of the acceleration process: the spectral shape fluctuations are of ~25% for both the maximum energy and the particle fluence.

Alvarez et al.^30^ demonstrated that the experimental energy distribution of the proton beam generated from laser acceleration is very similar to the proton burst produced in an ICF experiment using a shock ignition target of 48 MJ, in particular for the energy range of 2–6 MeV. We therefore ensured that our proton spectrum was containing these proton energies. All our materials were characterized before and after irradiation in order to verify the changes in the morphological, chemical, optical, electrical, and mechanical properties. Morphological information (i.e., surface roughness, presence of cracks, fractures, and holes after irradiation) of the surfaces was obtained by AFM and SEM microscopies while chemical composition of the surface was analyzed by energy dispersive X-ray (EDX) spectroscopy, taken under SEM conditions (see details in Supplementary Fig. 7).

## Discussion

We prepared the experimental setup and validated the experimental results using different Monte Carlo and energy deposition codes in which we inserted the same source parameters as obtained during the shots (see Fig. 1c, d). In order to have more reliable data, we used and compared three different codes: we performed simulations with the codes FLUKA^31,32^ and a customized energy deposition code that we had benchmarked with Geant4^33,34^, a well-known particle transport code used in the particle physics community (see Supplementary Fig. 4). The source given as input to those codes was modeled as the projection of a proton point source with energy-depending diverging rays, thus generating a laminar-diverging proton beam with a variable diameter at the source (see Fig. 1a, b), consistent with refs. ^1,2,35^ and measured data (see Fig. 1c, d). The simulation was using the quasi-Boltzmann spectral energy distribution as measured by the TPs during the experiment (see, e.g., Fig 1c) and that represents a typical spectrum obtained on such kind of high-power lasers^36–38^. For each material, the simulations provided the temperature and the released energy, specifying the contribution given by the electrons, ions, and photons.

In many facilities, a key parameter to monitor the deterioration of the materials is the displacements per atom (or dpa), a value that needs to be below a certain threshold value. To mention a few typical stress values on nuclear plants or ICF-MCF facilities, we can cite 15–30 dpa in a 5 years cycle^39,40^, with ~10 dpa maximum per full power year (fpy)^41^. We define the displacements per atom (or dpa) as the number of times that an atom is displaced for a given fluence, which is:

1 $${\mathrm{dpa}} = \varphi \sigma,$$

where *φ* is the beam fluence and *σ* is the cross-section of the process, i.e., the probability that the incident beam interacts with the matrix atoms. The fluence *φ* was evaluated using the proton beam spectrum that is irradiating the front surface of the target, such as obtained during the shots, as function of the proton energy. Considering the following formula, where *N*(*E*) is the measured proton spectrum (see Fig. 1c) and *A* the surface onto which the proton beam impinges, we obtain for the first surface layer using the TITAN laser:

2 $$\varphi = {\int} {\frac{{N\left( E \right)}}{A}} {\rm{d}}E = 3.2 \times 10^{17}{\mathrm{protons}} \times {\mathrm{m}}^{ - 2}.$$

For evaluating the irradiated material surface, we have used typical values obtained on the TITAN laser during the experiment, i.e., the divergence of the beam (see Fig. 1d) and the virtual source point (see Fig. 1e)—since the laser-driven proton source is divergent (but laminar) and distributed over a large surface (tens of µm diameter) the best way to model this kind of source is to consider that all particles are produced by a source point that is virtually located behind the real proton source (see Fig. 1b, e). Since the proton beam was stable within 25–30% shot-to-shot energy fluctuations, values within Fig. 1 can be assumed as a good representative for all the shots of the experiment.

Estimating the interaction cross-section *σ* for our materials^42^ to be in the order of 3 × 10^−25^ m^2^, we obtain *σφ* ~ 9.6 × 10^-8^ for one single shot on the TITAN laser. Considering the geometry of the experiment, the proton bunch impinging the material sample has a temporal length in the range of tens of ns. This is caused by the energy spread of the beam, which lengthens the proton beam from its ps length at the source to a few tens of ns at the moment when it reaches the material sample. Nevertheless, the bunch length is much shorter than what obtained on conventional facilities (usually ms). Using a proton bunch length of about *t* = 50 ns, and making the ratio with the value of *σφ*, we obtain a dpa/s value in the range of a few units. The high value of dpa/s is related to the extremely short duration of the impinging proton bunch (in the tens of ns range) and high charge (see Fig. 1c). However, we prefer to emphasize that the aim of our study is not to reproduce the dpa that are induced in facilities over a continuous timescale, but to analyze the overall damage caused by a single short proton shot, and compare it to existing methods. As such, while other techniques using longer irradiation times and low particles flux might allow for an easier relaxation of the material to be stress tested, the relevant result is the final damage provoked in the sample. In our case, this damage can be induced by one or, if needed, more shots, depending on the proton spectrum (indeed, the *σφ* value depends on the particle spectrum, which varies with laser energy). For simplicity, one can distinguish four different laser types that generate laser-driven protons. The first laser types are very high energy, longer pulse laser—currently difficult to obtain commercially such as the LLNL-TITAN laser (maximum energy: up to 180 J, typical pulse duration: 700 fs, central wavelength: 1.053 µm, repetition rate «1 Hz)^19,24^; the second types are high-energy laser, long-pulse laser—currently difficult to obtain commercially, but not out of reach for industry, such as the LULI-ELFIE (30 J, 350 fs, 1.056 µm, repetition rate «1 Hz)^43^; the third types are high-energy, short-pulse laser—similar to what can be obtained commercially as 1 PW laser (e.g., from Amplitude Technologies or Thales Optoelectronics), such as the ASTRA-GEMINI (10 J, 45 fs, 800 nm, envisioned rep-rate for future facilities 5–10 Hz (e.g., at the Extreme Light Infrastructure)^44^; and finally, the last types are high-energy, short-pulse laser—commercially available as 100–500 TW laser (e.g., from Amplitude Technologies) such as the ALLS or FZD-DRACO laser (5 J, 25 fs, 800 nm, rep-rate 10 Hz)^26,27^. Considering typical proton fluences on these facilities, one obtains, respectively, the following *σφ* values: *σφ* = 9.6 × 10^−8^, *σφ* = 1.5 × 10^−8^, *σφ* = 3 × 10^−9^, and *σφ* = 2.5 × 10^–9^. As can be seen, typical commercially available systems produce an about 30 times lower *σφ*; however, they have the advantage of being higher-repetition rate, which allows cumulating over several shots in order to produce the desired level of stress to the sample.

In order to confirm our calculations, we compute the *σφ* value using FLUKA and verify the induced temperature increase by simulating the energy deposition of the different particle species into the irradiated sample, according to the material’s stopping power. The numerical results for the different targets show for *σφ* a peak value (in the range of *σφ* = 2–3 × 10^−7^) within the first micron, then a rapidly decreasing value up to about 10 µm, before a slow decay phase starts, which brings the value from 1 × 10^−7^ down to 7 × 10^−8^ at the rear of the material target. The peak in the first micron of the target is due to the fact that higher-energy protons travel through the target and do not deposit most of their energy (Bragg peak) inside the bulk of the target, while lower energy get stuck in the first layers. Their contribution increases the global *σφ* value for the first layers.

Temperature values obtained with Geant4 and our custom-made code indicate that the temperature in the bulk heats up very quickly (consistently with typical proton-induced heating^45^), reaching its maximum temperature in the first ns and remaining constant before the cooling phase starts. The cooling phase when using laser-generated proton beams is shorter than what obtained on conventional stress tests facilities^46^ (where the cooling is in the ms regime for He and electron irradiation) and is in the order of tens of ns. Simulations confirm that during the entire process and for all materials listed in Table 1, the temperature within the sample stays safely below the melting point (about three times lower for the materials W, Ta, and C, see Table 1), therefore, the heating effect cannot strongly impact the properties of the analyzed samples.

**Fig. 2:**
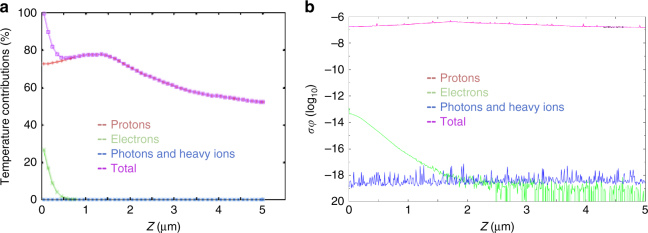
Temperature and *σφ* contributions for the different particle species. **a** Temperature and **b** *σφ* contributions of the laser-generated protons, electrons, photons, and heavy ions in a W target within the first 5 µm; the 0 level indicates the sample surface facing the impinging proton beam. Protons are indicated with a red line, electrons in green, and photons/heavy ions in blue. The total contribution is summarized with a purple line. Note that in plot **b**, the purple and red line fully overlap

Since the proton-generating target was unheated, protons were the most effectively accelerated particles^47^. However, in the plasma acceleration process, also other particles are accelerated and co-moving, these include mainly electrons, carbon ions from surface contaminants, gold particles (from the proton source target), oxygen ions, and photons^48^. Our Thomson Parabola was not able to detect neither traces of oxygen nor gold ions (see Supplementary Fig. 2), since their quantity was below the detection threshold (i.e., about four orders of magnitude lower than the proton signal, similarly as found in ref. ^49^). In order to estimate and validate the influence of these particles on the damage, we verified both the temperature influence on the global heating effect and their contribution to the global stress. The computed total deposited dose is indicated in Supplementary Fig. 5 and Supplementary Table 1; the simulations show that the influence of the heating by the electrons is lower than 20% in the first 500 nm, hence contributing very little, and then becomes completely negligible deeper in the target (see Fig. 2a). The photon and heavy ion (carbon, gold, and oxygen) heating contribution is always below 0.5%, therefore insignificant. Regarding the contribution to the total *σφ* value by the co-moving electrons and photons, we see that their contribution is completely negligible compared to the contribution produced by the protons^50^ (see Fig. 2b). Concerning the heavy ions co-moving with the proton beam (carbon, hydroxide, and gold), we consider that, as found in similar experiments^51^, their energy can generate a very widely distributed simple ion implantation on the target surface or sometimes produce a superficial coating effect (in particular the debris). However, their low fluence^48^ (below 10^10^ particles × MeV^−1^ × sr^−1^ for C and OH; 10^8^ particles × MeV^−1^ × sr^−1^ for gold) does not produce the growth of any carbon, oxygen, or gold monolayer on the target surface^52,53^. The ion implantation simply causes the formation of isolated defect points on the target surface, which can change the optical and electrical properties of the materials. We monitored these changes in the characterization of the materials, observing an opacification of the surface and the appearance of an optical band caused by the defective spots generated in the metallic lattice. In order to confirm that the protons, and not other particle species, provoked the damage, we repeated some shots using in front of the irradiated sample a 5 µm aluminum filter, able to stop all heavier ions and debris. Despite the filter, we still could find in the irradiated samples the same damage signature as found without the filter (see Supplementary Fig. 6).

**Fig. 3:**
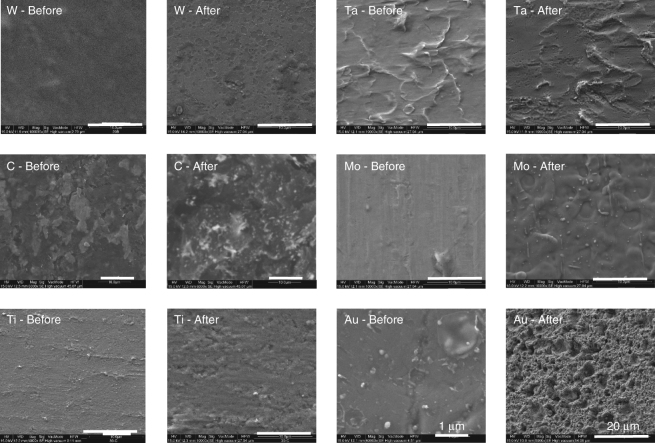
Morphological images. SEM images of all materials before and after proton irradiation. The bottom right white bar on all samples (except for the gold samples) indicates a 10 µm length. Gold images (bottom, right) have been reported for illustrating the effects of the proton irradiation on materials with low-melting point (~1065 °C for Au). For the gold sample, the scale before irradiation has been zoomed-in to 1 µm in order to check for surface details before irradiation and zoomed out to 20 µm in order to show the melting on a larger surface

The morphological analysis conducted by scanning electron microscope (SEM) (Fig. 3) reveals that after the proton irradiation, the initially smooth surface of the targets shows cracks, fractures, and holes indicating a strong surface erosion caused by the irradiation (few microns × shot^−1^ for W and C, and hundreds of nm × shot^−1^ for the other materials). Comparing our results to what obtained using conventional methods, and considering that we are particularly focusing on materials used in nuclear reactors, we see that our material exhibits very similar features to what found on a SEM image of W used as divertor for the DEMO Facility and loaded with hydrogen on a conventional facility (see ref. ^54^—the related image in the reference and our image display very similar craters and patches, a significant erosion and high void density). Similar features can also be found when comparing our results with what obtained using stress test based on conventional facilities using He (see ref. ^52^). None of our irradiated materials show melting regions or strong topographic changes on the surface. In comparison, a material with low-melting point such as gold, after the irradiation, shows a completely melted surface and the formation of a highly porous and disordered structure (see Fig. 3), with an erosion of hundreds of micron per  year. Morphological, mechanical, and optical characterizations have been obtained considering the proton spectrum as shown in Fig. 1. Since the proton spectrum tends to fluctuate during shots, the values of the characterization are subject to uncertainty, too, and depend on the delivered dose.

**Table 1:** Morphological, mechanical, and optical characterization

	Increase in surface roughness	Energy gap (eV)	Change in absorption within the range 400–700 nm (%)	Young’s modulus (GPa)	Variation in Young modulus	Stiffness (N/m)	Maximum sample temperature (°C)
Carbon	2%	0.6	0.17	53	87%	42.00	1340
Molybdenum	12.2%	1.5	27.2	13.5	88%	2.01	1820
Tantalum	11.3%	1.2	19.4	53.3	71%	68.00	2330
Titanium	9.5%	1.1	2.4	1	75%	1.00	1200
Tungsten	1.5%	0.7	0.16	163	50%	48.13	2380

Summary of the morphological, mechanical, and optical characterization of the considered materials. The optical absorption has been measured in the spectrum of the visible range

**Fig. 4:**
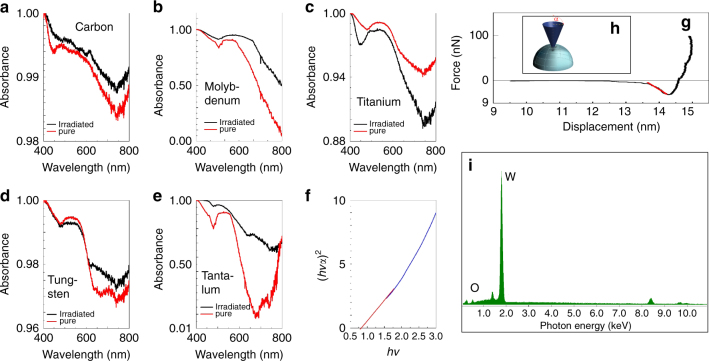
Additional material characteristics. **a**–**e** Optical absorption in the visible range for all target surfaces before (red line) and after (black line) proton irradiation; **f** Tauc plot and **i** EDX analysis after irradiation for W as example for all materials; **g** Tip force as function of piezo displacement for indentation measurements for the tungsten sample as example for all materials (the red line is for estimating the Young’s modulus); **h** Sketch of the theoretical model used to study the interaction between the conical tip and the material in the scanning model used in this paper

Optical absorption measurements in Fig. 4a–e show a general increase in the optical absorption with greater values for Mo and Ta (19.4% and 27.2%, respectively). This corresponds to an increase in the band gap (see Tauc’s plot for W as example for all materials in Fig. 4f), which ranges from the 0.6 eV of carbon to the 1.5 eV of molybdenum, suggesting a formation of a small layer of oxide on the material surface (we hypothesize a thickness of a few nanometers). The changes in the optical gaps can be associated to both changes in the surface roughness and a large amount of local defects/gaps introduced in the material’s lattice by the proton irradiation, defects that change the density of state in the irradiation points. This results in strong changes of the electronic properties, the loss of a metallic behavior, and the appearance of an increasing energy gap. AFM measurements (see details in the Supplementary Note 1) indicate a general increase of the surface roughness of about 10% for all materials while nano-indentation measurements under AFM conditions (see Table 1 and Fig. 4g, a sketch of the theoretical model used to study the interaction between the conical tip and the material in the scanning model is displayed in Fig. 4h) indicate a general decrease of the Young’s modulus and a consequent increase in the stiffness, which suggests a general increase in the target rigidity, ranging from 50% for molybdenum up to 87% for tungsten.

EDX analysis under SEM conditions (see Fig. 4i for the material W as example for all materials) indicates that the chemical composition of the target materials is unchanged within the detection limit of the EDX (1000 ppm), with only a small weight percentage presence of gold (~3%), indicating the implantation of energetic gold atoms (present in the proton beam residuals of the TNSA mechanism) on the target surface, and a small amount of oxygen detected into the first surface layers. The non-negligible gold ion implantation on the sample materials suggests that it is possible to implant energetic atomic and ion beams produced during the nuclear fusion process and to induce strong chemical changes on the material surface. The small oxygen amount can be attributed to the oxygen impurities in the proton beam: during the acceleration process, a few oxygen atoms are stemming out from the back surface of the target. These atoms are coming from a very thin contaminant layer located on the back target surface (an example of detailed composition of the back surface can be found in refs. ^55^ and ref. ^56^, mentioning a 12–20 Å-thick layer consisting of 27% gold, 60.5% hydrocarbons (CH_2_), and 12.2% water vapor (H_2_O)). From the EDX microanalysis, we can estimate the oxygen percentage to be in the order of 5% (Fig. 4i).

### Data availability

All the data are available from the corresponding author on reasonable request.

## Electronic supplementary material

Supplementary Information
