# Publisher Correction: Laser-accelerated particle beams for stress testing of materials

**DOI:** 10.1038/s41467-018-04345-y

**Published:** 2018-05-14

**Authors:** M. Barberio, M. Scisciò, S. Vallières, F. Cardelli, S. N. Chen, G. Famulari, T. Gangolf, G. Revet, A. Schiavi, M. Senzacqua, P. Antici

**Affiliations:** 1 INRS-EMT, 1650 Boul. Lionel Boulet, Varennes, QC Canada; 2 University of Rome “La Sapienza”, Dip. SBAI and INFN, Via A. Scarpa 16, 00161 Roma, Italy; 3 LULI, Ecole Polytechnique, Route de Saclay, 91128 Palaiseau, France; 4 Institute of Applied Physics, 46 Ulyanov Street, 603950 Nizhny Novgorod, Russia; 5 Medical Physics Unit, McGill University, Montreal, QC Canada

Correction to: *Nature Communications* 10.1038/s41467-017-02675-x, published online 25 January 2018

The original version of the Supplementary Information associated with this Article contained an error in Supplementary Figure 3, in which all panels, with the exception of the bottom-left ‘Ti’ panel, were blank. The HTML has been updated to include a corrected version of the Supplementary Information.

