# Supplementary material for: Laser-accelerated particle beams for stress testing of materials
Source: Nat Commun. 2018 Jan 25;9:372. doi: 10.1038/s41467-017-02675-x (PMC5785512; doi:10.1038/s41467-017-02675-x)
Supplement: Supplementary file 1 — Supplementary Information [file 41467_2017_2675_MOESM1_ESM.pdf]

Supplementary Note 1: Details about the material science diagnostics

AFM images were obtained using a Bruker-ICON AFM microscope working in tapping mode. Each image was taken with a resolution of 512x512 pixels and a frequency of 1 Hz.

The nano-indentation analysis for measuring the changes in the mechanical properties module was performed under AFM condition, using a Sneddon model (conical indenter)[1]. The optical absorption of the particle films was measured under an Olympus microscope (Horiba-Jobin-Yvon) equipped with a white lamp, and with a Triax 320 spectrometer working in the 200-1500 nm range. We measured directly the reflected spectrum $I_r(\lambda)$and obtained, assuming the transmittance to be zero for the bulk samples, the reflectance $r(\lambda)$and the absorbance $\alpha(\lambda)$as a function of wavelength by the relation:

$$r(\lambda) = \frac{I_r(\lambda)}{I_s(\lambda)} \quad (1)$$

$$\alpha(\lambda) = (1 - r(\lambda)) \quad (2)$$

where $I_s(\lambda)$is the source spectrum. The energy gap of the materials after irradiation was obtained from optical absorption using the Tauc's model. We used the value r= ½ for the exponent in the plot of $(\alpha h\nu)^{1/r}$ as function of $h\nu$, denoting the nature of direct transitions of the observed phenomena[2].

Supplementary Note 2: Details about the Thomson Parabolas

As proton diagnostic we used two calibrated Thomson Parabolas (TPs) located at 0° (TP 0°) and 16° (TP 16°) with respect to the main pulse laser axis to measure the forward generated proton spectrum. The TPs were placed respectively at a distance of 690 and 565 mm from the proton source (distance to the entrance slit). The magnetic field of the dipole for both TPs was ∼500 mT and had a length of 150 mm. The voltage applied to the electrodes following the magnetic dipole was +-3 kV. The incoming ion beam at the entrance of the TP was selected with an aperture of ∼0.5 mm. Proton spectra measured by the TPs were readout in an absolute manner[3, 4] using Image Plates (BAS-TR 2025 from Fuji Photo Film Co. Ltd) that were analyzed using a FUJIFILM FLA-7000 reader (see example of a raw image in Supplementary Figure 1). Additional measurements of the proton spectra were obtained using Radio Chromic Films (RCFs) of the type HS that allowed obtaining a beam spatial distribution. The calibration was performed by using known metallic foils of Al, placed in front of the Image Plates, that produce well defined cut-off energies and are linked to a specific distance from the beam center (also called 0 point). These cut-off distances allow the reconstruction of a curve showing deflections vs particle energy, which is the calibration of the specific TP.

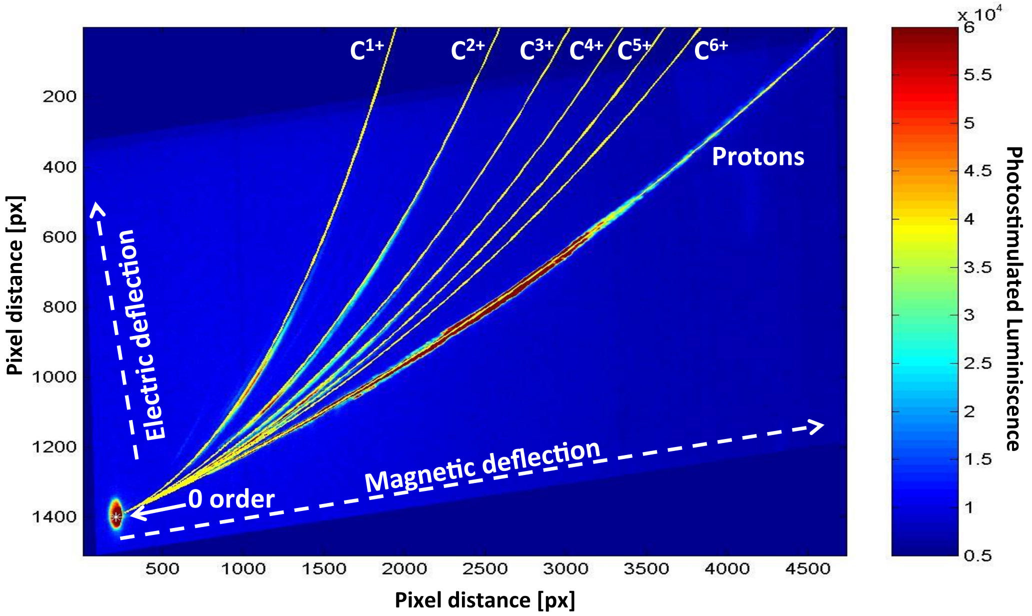

Supplementary Figure 1 - Raw image of the Image Plate as obtained by a Thomson Parabola. Raw Image Plate image as obtained by the Image Plate diagnostics of the Thomson Parabola, displaying the proton and carbon spectra. The yellow lines that follow the carbon and proton traces are a guide for the eye and have been computed with analytical formulas using the Thomson Parabola parameters.

Supplementary Figure 1 shows the raw data as measured by the Image Plate of a TP located at 0° and recording the spectra obtained on the laser facility when using a Gold foil target with a thickness of ~10 µm. One can identify the proton trace and the traces related to other ion species as indicated. The horizontal deflection is due to the electric field in the parabola, the horizontal deflection is caused by the magnetic field in the TP.

Supplementary Figure 2 shows an example of a deconvoluted carbon spectra as obtained using a solid 10 µm gold target. Compared to the number of protons reported in fig. 1.D of the main manuscript, the number of carbon ions is significantly less (there is a difference of more than two orders of magnitude) for all energies within the spectrum of each species.

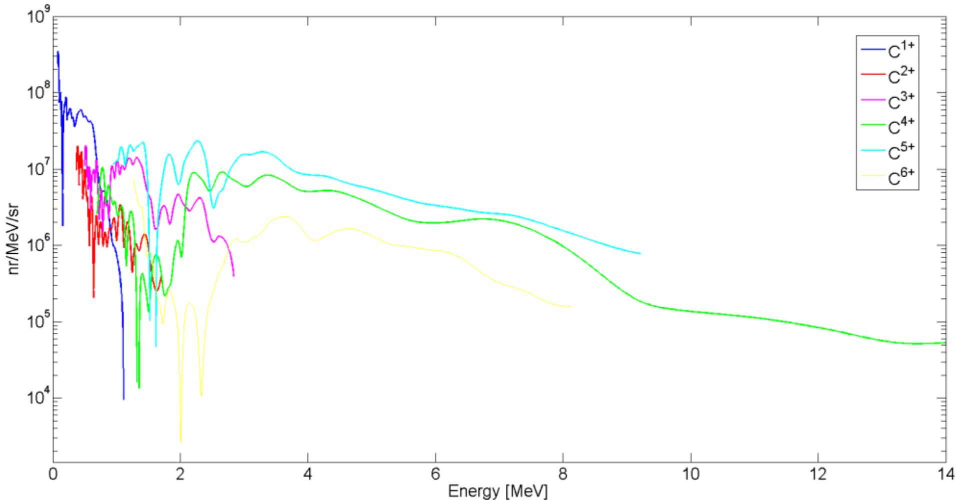

Supplementary Figure 2 - Deconvoluted carbon spectra. Carbon spectra as obtained from the Image Plate shown in Figure 1.

Supplementary Note 3: Maximum temperature as obtained during the laser-driven proton irradiation

Supplementary Figure 3 shows the temperature maps for the material samples used in the current experiment as obtained with our custom code that has been benchmarked with other Monte Carlo codes used in the community such as FLUKA and Geant4. The radial distance of R=0 corresponds to the proton beam center. Since the samples were placed at 1 cm above beam center, the temperature to consider for the sample is the temperature at R=1 cm (the temperature below R=1 cm has been added for general information). We add radial and longitudinal lineouts for the tungsten sample (as example for all) to show the radial and longitudinal evolution of the temperature within a sample in Supplementary Figure 4.

In our case, the heating of the samples to temperatures in the 2500°C occurs over very short timelines (in the tens of ns at maximum). Therefore temperature measurements using pyrometers or thermocouples cannot resolve the quick heating phase. Spectrometer measurements, such as x-ray absorption near edge spectroscopy (XANES)[5], would be more appropriate for these timescales, but the precision of this measurement technique is not optimized for our experiment.

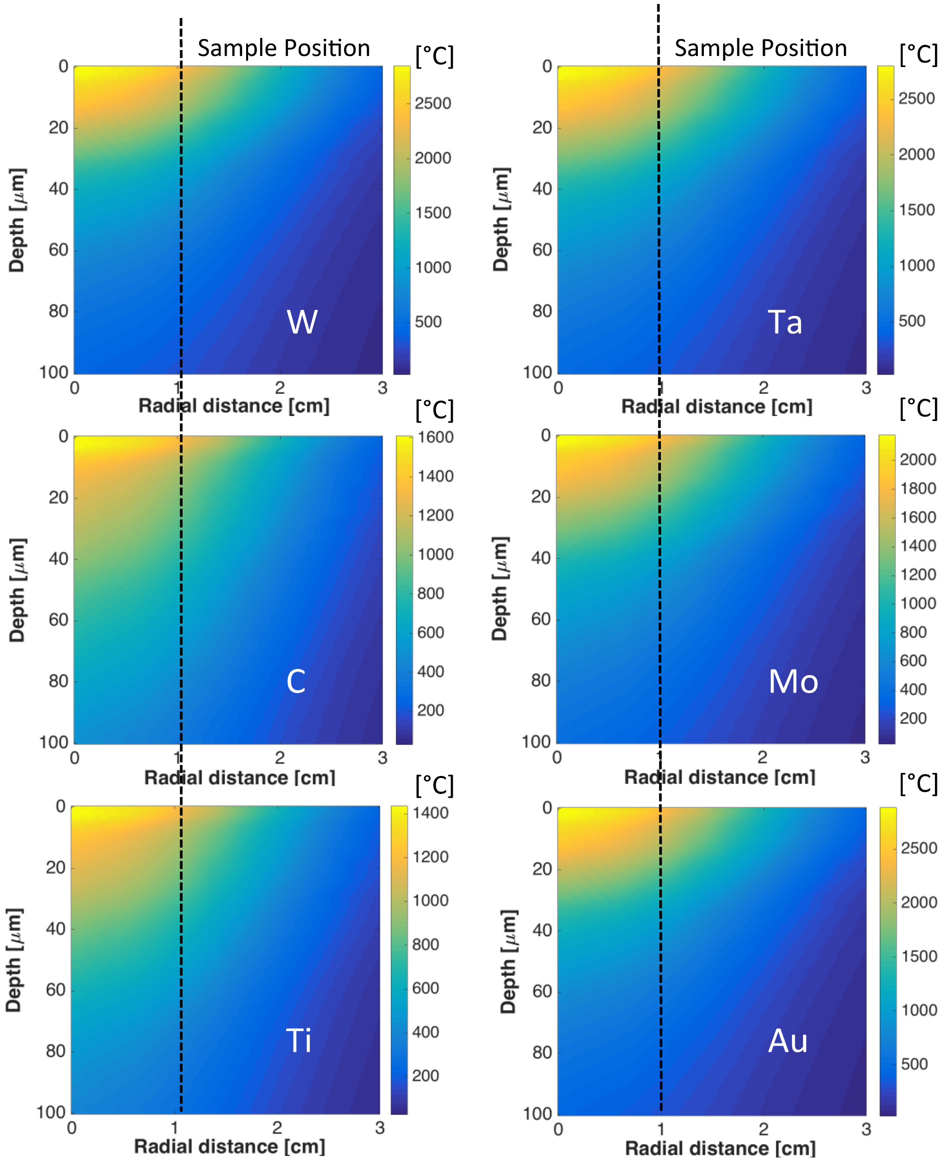

Supplementary Figure 3 - Temperature maps for the different samples used in the present study. Different temperatures maps obtained by the Energy Deposition Code for the different samples used in the present study. The line indicates the position R=1 where the sample was placed. The protons are impinging the samples from the top. The materials are labeled on the temperature maps in white.

Supplementary Note 4: Benchmarking of different codes to verify the energy deposition

In Supplementary Figure 4 we show the results of the benchmarking between our energy deposition code and Geant4. Regarding Geant4, we used the version 10.2 (patch 02) and the physics list G4EmStandardPhysics_option4, the secondary particle production cut off was 10 nm. In both cases, the simulation engines were taking as input data the proton spectrum, the cone beam half-angle as well as the virtual source position

variation with proton energy. Concerning the custom-made code, proton energy is deposited locally through the use of stopping power values for each material available on the NIST-PSTAR database[6]. The full cone beam is split in multiple beamlets with different incident angles on the target. A Gaussian transverse proton beam intensity profile is used, for which the angular standard deviation corresponds to the cone beam's half-angle. Supplementary Figure 4.a shows the temperature map as obtained with the Geant4 code for the tungsten sample in the same conditions as for Supplementary Figure 3 (upper left temperature map). The absolute difference, in percentage, is indicated in Supplementary Figure 4.b, displaying negligible temperature variations (<5%) in the regions of interest of the sample. Supplementary Figure 4.c shows a depth lineout of the temperature maps for the tungsten sample at the radial position R=0, while Supplementary Figure 4.d the radial lineout at the surface (depth=0 µm). Lineouts are taken for both, the Geant4 and our energy deposition code simulations.

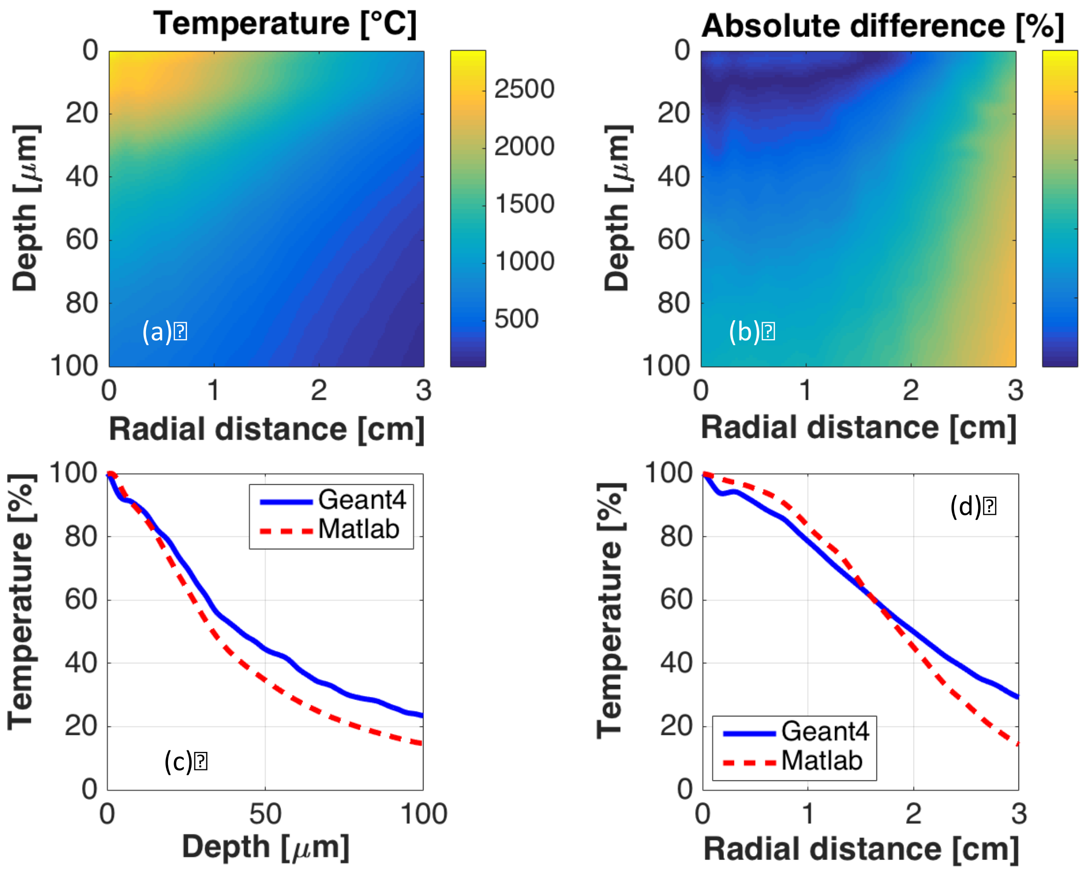

Supplementary Figure 4 – Temperature maps for our Energy Deposition Code and Geant4. (a) Temperature map for the tungsten sample as computed by the Geant4 code; (b) Absolute difference between the computed values as obtained with our custom-made code and the Geant4 code; (c) depth lineout of both maps for R=0 cm; (d) radial lineout at the surface.

Supplementary Note 5: Dose deposition

The calculated dose, indicated in J/kg, depends on the volume to be considered. Due to the large energy spectrum, the impinging particles deposit their dose in different depths. The table below gives information about the deposited dose as calculated by the Energy Deposition code for several relevant volumes (see figure below for the different volumes). The average dose D_{ave} is computed as follows: $D_{ave}= c_p * \Delta T_{ave}$

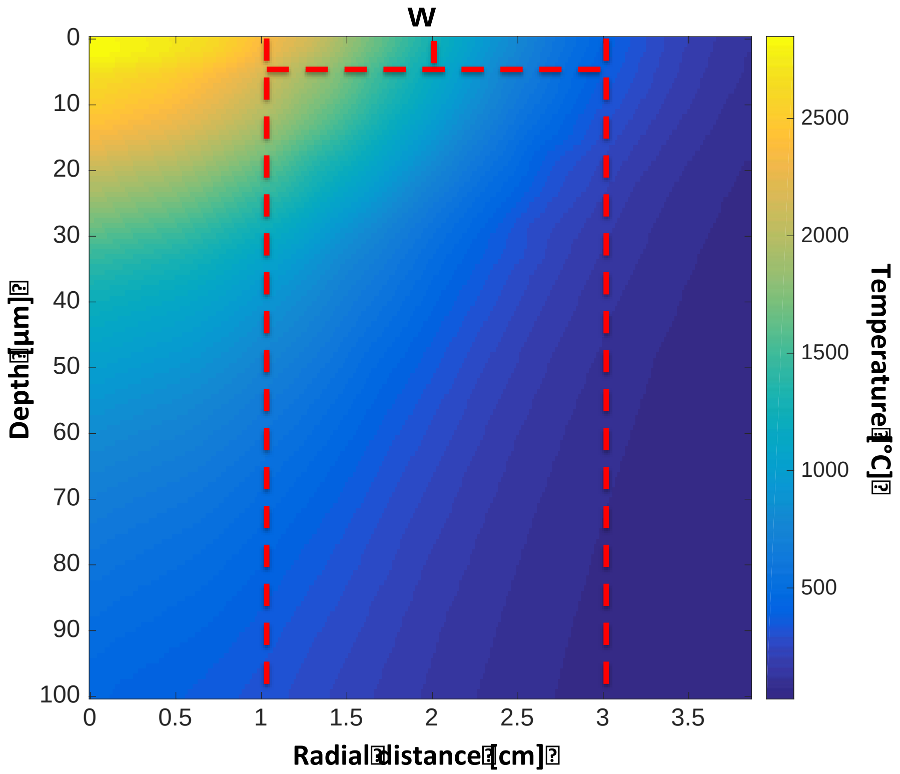

Supplementary Figure 5 - Schematics for the dose calculations. Schematics of how the dose calculations have been made: The sample is located within the Radial distance 1-3 cm. Different depths are considered which produce different volumes.

The considered volumes are
V_1=2 mm x 20 mm x 100 µm
V_2=2 mm x 10 mm x 100 µm
V_3=2 mm x 20 mm x 5 µm
V_4=2 mm x 10 mm x 5 µm

Material	c_p [J/kg/°C]	$\Delta T_{1,ave}$ [°C]	$D_{1,ave}$ [kGy]	$\Delta T_{2,ave}$ [°C]	$D_{2,ave}$ [kGy]	$\Delta T_{3,ave}$ [°C]	$D_{3,ave}$ [kGy]	$\Delta T_{4,ave}$ [°C]	$D_{4,ave}$ [kGy]
Au	**129**	500	**64.5**	1271	**164**	721	**93.0**	1779	**229**
Graphite	**720**	374	**269**	701	**505**	534	**384**	983	**708**
Mo	**250**	405	**101**	945	**236**	582	**146**	1324	**331**
Ta	**140**	508	**71.2**	1235	**173**	732	**102**	1728	**242**
Ti	**523**	315	**165**	626	**327**	451	**236**	877	**459**
W	**132**	491	**64.8**	1253	**165**	708	**93.4**	1754	**232**

Supplementary Table 1 - Different doses as calculated using the Energy deposition code.

Supplementary Note 6: Protected sample images

Scanning Electron Microscope (SEM) image of tungsten sample protected by a 5 µm aluminum foil and irradiated with the proton beam generated on the TITAN laser.

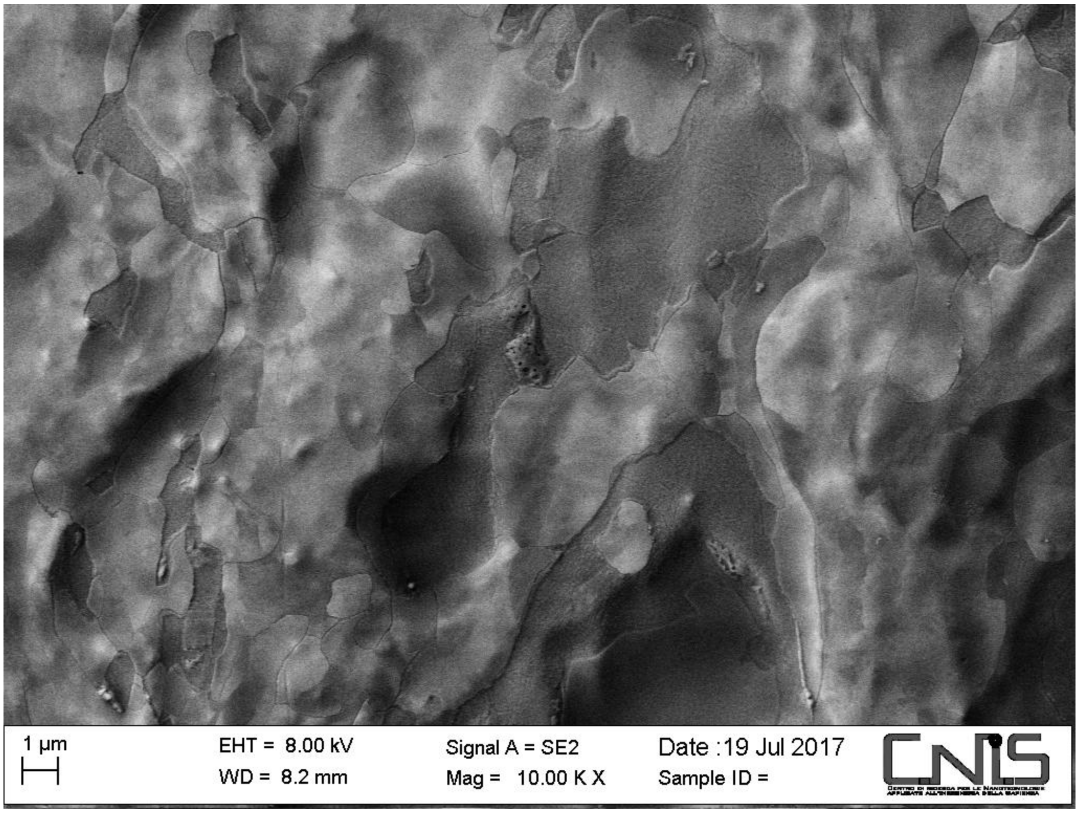

Supplementary Figure 6 - SEM image of a Tungsten sample protected by a 5 µm Al foil. SEM image of a Tungsten sample irradiated by laser-generated protons as obtained on the TITAN laser. The Tungsten sample has been screened by a 5 um aluminum foil in order to avoid contributions by heavier ions.

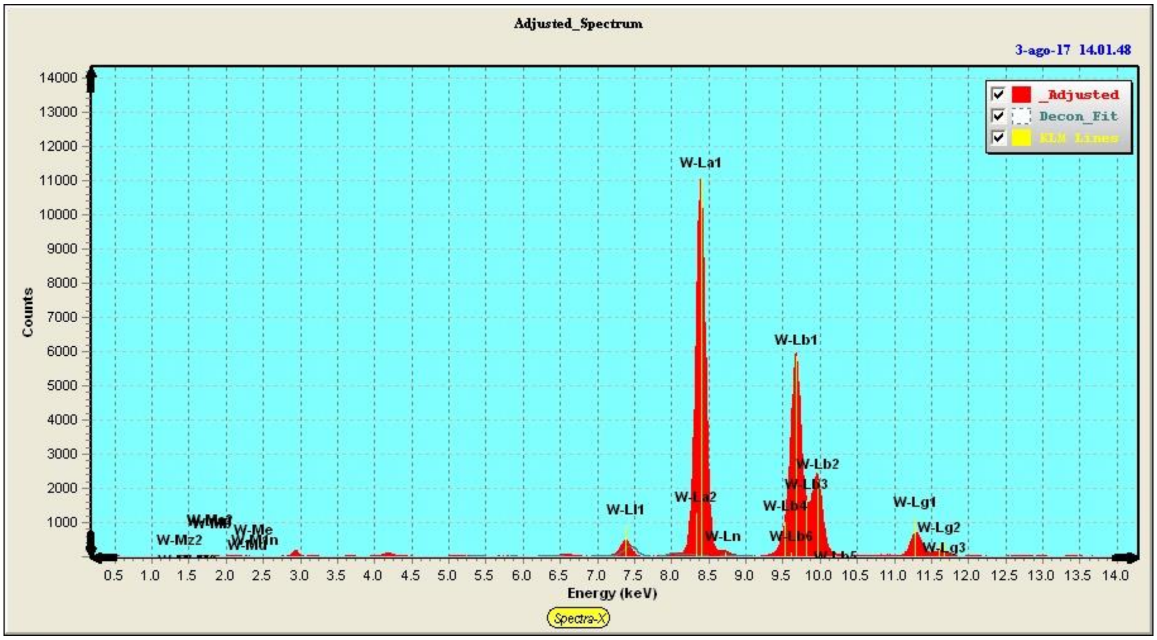

Supplementary Figure 7 - XRF analysis of the tungsten sample. XRF analysis of the tungsten sample irradiated with laser-generated proton. The tungsten foil that has been protected with a 5 µm thick aluminum foil.

Supplementary References

[1] Reynaud, C., Sommer, F., Quet, C., El Bounia, N., and Minh Duc, T., Quantitative determination of Young's modulus on a biphase polymer system using atomic force microscopy, *Surface and Interface analysis* **30**, 185-189 (2000)

[2] Davis, E.A., Mott, N. F., Conduction in non-crystalline systems V. Conductivity, optical absorption and photoconductivity in amorphous semiconductors, *Philosophical Magazine A* **22** (179): 903-922 (1970)

[3] Mančić, A., Fuchs, J., Antici, P., Gaillard, S. A., and Audebert, P., Absolute calibration of photostimulable image plate detectors used as (0.5-20 MeV) high-energy proton detectors, *Rev. Sci. Intrum*. **79**, 073301-073306 (2008)

[4] Bonnet, T. et al., Response functions of Fuji imaging plates to monoenergetic protons in the energy range 0.6–3.2 MeV, *Rev. Sci. Instrum.* **84**, 013508-013513 (2013)

[5] Mančić, A. et al., Picosecond Short-Range Disordering in Isochorically Heated Aluminum at Solid Density, *Phys. Rev. Lett.* **104**, 035002 (2010)

[6] http://physics.nist.gov/PhysRefData/Star/Text/intro.html (accessed 6 October 2017)
